# Supplementary material for: Area Wide Monitoring of Plant and Honey Bee (Apis mellifera) Viruses in Blueberry (Vaccinium corymbosum) Agroecosystems Facilitated by Honey Bee Pollination
Source: Viruses. 2023 May 20;15(5):1209. doi: 10.3390/v15051209 (PMC10220920; doi:10.3390/v15051209)
Supplement: Supplementary file 1 [file viruses-15-01209-s001.zip › viruses-2329810-Supplementary Tables.pdf]

Table S1. Blueberry shock virus, prune dwarf virus, and black queen cell virus coat and capsid protein nucleotide sequence genbank accession numbers and number of mapped reads.

Sample	RNAseq file	Sample type	Mapped reads	Genbank accession
Blueberry shock virus coat protein				
BCFV1-BB-4B	21BP028	Bread	1346	OQ957511
BCFV2-BB-1B	21BP029	Bread	605	OQ957513
BCFV2-BB-2B	21BP030	Bread	758	OQ957514
BCFV2-BB-3B	21BP031	Bread	646	OQ957515
BCFV2-BB-1F	21BP005	Forager bee	691	OQ957516
BCFV2-BB-2F	21BP006	Forager bee	1052	OQ957517
BCFV2-BB-3F	21BP007	Forager bee	378	OQ957518
BCFV2-BB-2H	21BP014	Hive Bee	143	OQ957520
BCFV2-BB-2P	21BP038	Pollen	335	OQ957523
BCFV2-BB-3P	21BP039	Pollen	127	OQ957524
BCFV2-BB-4P	21BP040	Pollen	68	OQ957525
Prune dwarf virus coat protein				
BCFV1-BB-2B	21BP026	Bee Bread	111	OQ957526
BCFV1-BB-3B	21BP027	Bee Bread	860	OQ957527
BCFV1-BB-4B	21BP028	Bee Bread	3685	OQ957528
BCFV1-BB-1H	21BP009	Hive Bee	178	OQ957529
BCFV1-BB-2H	21BP010	Hive bee	185	OQ957530
BCFV2-BB-2B	21BP030	Bee bread	1575	OQ957531

BCFV2-BB-4B	21BP032	Bee Bread	271	OQ957532
BCFV2-BB-1B	21BP029	Bee Bread	4720	OQ957533
BCFV2-BB-4F	21BP008	Forager bee	146	OQ957534
BCFV2-BB-4H	21BP016	Hive Bee	82	OQ957535
Black queen cell virus capsid protein 4				
BCFV1-BB-1F	21BP001	Forager bee	14438	OQ957536
BCFV1-BB-2F	21BP002	Forager bee	334	OQ957537
BCFV1-BB-4F	21BP004	Forager bee	9904	OQ957538
BCFV2-BB-1F	21BP005	Forager bee	18080	OQ957539
BCFV2-BB-2F	21BP006	Forager bee	16477	OQ957540
BCFV2-BB-3F	21BP007	Forager bee	7877	OQ957541
BCFV1-BB-1H	21BP009	Hive Bee	16360	OQ957542
BCFV2-BB-1H	21BP013	Hive Bee	13911	OQ957543
BCFV2-BB-2H	21BP014	Hive Bee	5938	OQ957544
BCFV2-BB-3H	21BP015	Hive Bee	12287	OQ957545
ON1-BB-1F	21BP017	Forager bee	49	OQ957546
BCFV2-BB-2P	21BP038	Pollen	120	OQ957547

Table S2: Sequence read archive accession numbers for RNAseq files

Accession	Sample Name
SAMN34718726	BCFV1-BB-1B
SAMN34718727	BCFV1-BB-2B
SAMN34718728	BCFV1-BB-3B

SAMN34718729	BCFV1-BB-4B
SAMN34718730	BCFV1-BB-1F
SAMN34718731	BCFV1-BB-2F
SAMN34718732	BCFV1-BB-3F
SAMN34718733	BCFV1-BB-4F
SAMN34718734	BCFV1-BB-1H
SAMN34718735	BCFV1-BB-2H
SAMN34718736	BCFV1-BB-3H
SAMN34718737	BCFV1-BB-4H
SAMN34718738	BCFV1-BB-1P
SAMN34718739	BCFV1-BB-2P
SAMN34718740	BCFV1-BB-3P
SAMN34718741	BCFV1-BB-4P
SAMN34718742	BCFV1-BB-T1
SAMN34718743	BCFV1-BB-T2
SAMN34718744	BCFV2-BB-1B
SAMN34718745	BCFV2-BB-2B
SAMN34718746	BCFV2-BB-3B
SAMN34718747	BCFV2-BB-4B
SAMN34718748	BCFV2-BB-1F
SAMN34718749	BCFV2-BB-2F
SAMN34718750	BCFV2-BB-3F
SAMN34718751	BCFV2-BB-4F
SAMN34718752	BCFV2-BB-1H
SAMN34718753	BCFV2-BB-2H
SAMN34718754	BCFV2-BB-3H
SAMN34718755	BCFV2-BB-4H
SAMN34718756	BCFV2-BB-1P
SAMN34718757	BCFV2-BB-2P
SAMN34718758	BCFV2-BB-3P
SAMN34718759	BCFV2-BB-4P

SAMN34718760	BCFV2-BB-T1
SAMN34718761	BCFV2-BB-T2
SAMN34718762	ON1-BB-1B
SAMN34718763	ON1-BB-2B
SAMN34718764	ON1-BB-3B
SAMN34718765	ON1-BB-1F
SAMN34718766	ON1-BB-2F
SAMN34718767	ON1-BB-3F
SAMN34718768	ON1-BB-1H
SAMN34718769	ON1-BB-2H
SAMN34718770	ON1-BB-3H
SAMN34718771	ON1-BB-1P
SAMN34718772	ON1-BB-2P
SAMN34718773	ON1-BB-3P
SAMN34718774	ON1-BB-1T1
SAMN34718775	ON1-BB-1T2

Table S3. Bee viruses detected in leaf and flower samples from two BC blueberry farms.

Bee virus	Genus	BC site 1	BC site 2	Average frequency of detection (%)	Average genome coverage (%)	Average VRPM
		Plant tissue	Plant tissue			
		n=2	n=2			
		Frequency				
Lake Sinai Virus	Sinaivirus	100	100	100	56.6	12

Table S4. Bee viruses detected in leaf and flower samples from on ON blueberry farm.

Bee virus	Genus	ON site 1	Average frequency of detection (%)	Average genome coverage (%)	Average VRPM
		Plant tissue			
		n=2			
		Frequency			
Lake Sinai Virus	Sinaivirus	100	100	88.6	236
Black Queen Cell Virus	Triatovirus	50	50	17.8	32
